# Statistical issues in the use of dynamic allocation methods for balancing baseline covariates

**DOI:** 10.1038/bjc.2011.157

**Published:** 2011-05-03

**Authors:** G R Pond

**Affiliations:** 1 Department of Oncology, McMaster University, Ontario Clinical Oncology Group, Research Centre at Juravinski Hospital, G(60) Wing, 1st Floor, 711 Concession Street, Hamilton, ON, L8V 1C3 Canada

**Keywords:** dynamic allocation, minimisation, balancing baseline covariates

## Abstract

**Background:**

The procedure for allocating patients to a treatment arm in comparative clinical trials is frequently chosen with only minor deliberation. This decision may, however, ultimately impact the trial inference, credibility, and even validity of the trial analysis. Cancer researchers are increasingly using dynamic allocation (DA) procedures, which balance treatment arms across baseline prognostic factors for clinical trials in place of historical methods such as simple randomisation or allocation via the random permuted blocks.

**Methods:**

This article gives an overview of DA methods, the statistical controversy that surrounds these procedures, and the potential impact on a clinical trial results.

**Results:**

Simple examples are provided to illustrate the use of DA methods and the inferential mistakes, notably on the *P*-value, if incorrect analyses are performed.

**Interpretation:**

The decision about which method to use for allocating patients should be given as much consideration as other aspects of a clinical trial. Appropriately choosing between methods can affect the statistical tests required and what inferences are possible, while affecting the trial credibility. Knowledge of the different methods is key to appropriate decision-making.

Randomised clinical trials have long been considered the gold standard in clinical research (Concato *et al*, 2000). Strictly speaking, a randomised clinical trial is one where the allocation of patients to treatment arm occurs according to a random mechanism. In practice, this is typically performed using some sort of computer-generated list or random number generator. The allocation procedure is termed simple random sampling (Zelen, 1974) and gives every patient the exact same chance of being allocated to receive each treatment. The use of a random mechanism is the cornerstone of these trials and is the basis for statistical theory and analysis of these trials (Greenland, 1990). However, not all clinical trials use a strict randomisation procedure to allocate patients. Dynamic allocation (DA) methods (Pocock and Simon, 1975), which balance prognostic factors between treatment groups, often referred to as minimisation (Taves, 1974), are a primarily deterministic, non-random algorithm being implemented with increasing regularity by cancer researchers in clinical trials (Pond *et al*, 2010). The effect on clinical trial interpretation when using these methods is not necessarily trivial, and has caused substantial debate regarding their usefulness and validity (Senn, 2000). Some authors argue that for clinical trials, ‘if randomisation is the gold standard, minimisation may be the platinum standard’ (Treasure and MacRae, 1998). Other authors have claimed these techniques are not necessary, possibly even detrimental, and use of these methods should be ‘strongly discouraged’ (Committee for Proprietary Medicinal Products, 2003; Senn, 2004).

While this controversy appears to be well discussed in the statistical literature (Rosenberger and Lachin, 2002; Buyse and McEntegart, 2004a; Roes, 2004; Taves, 2004; Day *et al*, 2005; Senn *et al*, 2010), anecdotally it appears less appreciated in the clinical cancer research literature. Further, and possibly of greater concern, is there may be an underappreciation of the effect of non-randomised allocation on results, including the *P*-value, when using standard statistical analyses. Therefore, this manuscript was written with an aim to inform investigators who plan to incorporate DA methods in their clinical trials of some of the strengths and limitations of these techniques.

## What is dynamic allocation?

Randomisation permits an unbiased comparison between patients allocated to different treatments (Altman and Bland, 1999). Use of randomisation ensures *asymptotic* balancing of patients to treatment, and of baseline prognostic factors, including factors that are unknown at the time of randomisation. That is, the number of patients allocated to each treatment arm will approach equality, and prognostic factors will be equally balanced within patients across different treatments, in a clinical trial *as the sample size increases infinitely*. For any given trial, which has a *finite* sample size, however, there may be an imbalance between treatment arms in one, or more, known or unknown prognostic factors. Standard statistical theory is able to objectively quantify the possibility of an imbalance and even correct or adjust results for imbalances when they do occur (Lachin, 1988). However, performing statistical adjustments for imbalanced prognostic factors are not recommended if the adjustment was not initially planned as this can lead to questions of multiple testing (Pocock *et al*, 1987). Even when adjusting for an imbalanced prognostic factor is planned initially, a clinical trial having an imbalance, especially a large one, can be very concerning and possibly even detrimentally affect the credibility of the trial (Buyse and McEntegart, 2004b). Methods that reduce the possibility of a trial having a large imbalance between treatment arms for a known prognostic factor have therefore been proposed. Taves (1974) proposed an algorithm called minimisation, which is a deterministic method to allocate patients to treatment. The following year, Pocock and Simon (1975) independently presented a more general family of algorithms, called DA methods of which minimisation is one specific approach. Although earlier reviews indicated these methods were used very infrequently (Altman and Dore, 1990; Lee and Feng, 2005), a recent cancer-specific review of multi-arm clinical trials indicated their use is increasing and no longer uncommon (Pond *et al*, 2010).

To illustrate how DA methods work, let us consider a hypothetical two-arm clinical trial in breast cancer where three patient baseline covariates are considered prognostic: Her2-neu status (positive or negative), menopausal status (post-menopausal or pre-/peri-menopausal), and stage of disease (II or III). Assume the breakdown by treatment of the baseline prognostic factors for the first 19 patients is summarised in Table 1 and the 20th patient, a post-menopausal, Her2-neu-negative patient with stage II disease, is ready to be enrolled in the trial.

Using Taves minimisation algorithm, or equivalently the Pocock–Simon range method with allocation probability of 1, the number of previously enrolled patients with the same prognostic factor as the new patient is counted. The new patient would then be allocated to the treatment arm for which the sum of the previously enrolled patient prognostic factor counts is smallest. That is, if the 20th patient was allocated to treatment A, then there would be 5+1 Her2-neu-negative patients, 6+1 post-menopausal patients, and 7+1 stage II patients assigned to treatment A, which is summed to be (5+1)+(6+1)+(7+1)=21. Alternatively, if the 20th patient was allocated to treatment B, a total sum of (3+1)+(4+1)+(2+1)=12 is obtained. Since 12<21, the patient would be assigned to treatment B, minimising the imbalance.

Many authors have proposed, and continue to propose, modifications to these algorithms (Wei, 1977; Begg and Iglewicz, 1990; Heritier *et al*, 2005; Perry *et al*, 2010). While some of these methods may modestly increase efficiency, most are rarely used. One modification that does appear to be utilised regularly is to add in a random component; thus, allocation is no longer completely deterministic. For example, in the hypothetical trial above, patient 20 would be allocated to treatment B with probability *P*, 0.5<*P*<1. A probability of *P*=0.8 has been shown to be most efficient (Brown *et al*, 2005).

## Other common allocation methods

Simple random sampling is the most basic allocation method (Zelen, 1974). Each patient is assigned with equal probability to different treatment arms, regardless of all other considerations. Generally performed in practice by creating a randomisation list based on a random number table, or a computerised random number generator, it is equivalent to the notion of allocating patients by flipping a fair coin.

Another frequently used method is allocation via the random permuted blocks (Zelen, 1974). Also known as block randomisation, stratified random sampling, or permuted block sampling, the patient allocation list is grouped into blocks of size 2*k* (assuming 1 : 1 allocation) with *k* patients in each block assigned to each treatment. For example, if the block size is set to 4, then there are six ways, or permutations, in which two patients can be allocated to each treatment within a given block: AABB, ABAB, ABBA, BAAB, BABA, and BBAA. For each block, a permutation is selected at random and patients are assigned to treatment as they are enrolled according to that permutation. When that block is full, another permutation is selected for the next group of patients. A separate list of permuted blocks is created for each combination of strata. In the illustrative example, there would be 2 × 2 × 2=8 lists created, one for each combination of Her2-neu status, menopausal status, and disease stage. Although not well understood, allocation via the random permuted blocks method is not an entirely random technique. The last patient allocated within each block is completely determined by the allocation of the previous patients within that block. To reduce potential selection bias, a simple modification is to vary the block size throughout the trial.

Another dynamic procedure is a biased coin method (Efron, 1971). Using this method, a patient is randomly allocated to the treatment arm which has fewer patients already accrued with probability *P*, where 0.5<*P*<1. If there is no difference in the number of patients treated in each arm, the next patient has an equal probability of being assigned to each treatment. An adaptive biased coin design (Hofmeijer *et al*, 2008) is one where the value of *P* for each patient allocation depends on the degree of imbalance in the number of patients previously enrolled to each arm. Alternatively, one could perform simple random sampling as long as the imbalance in the number of patients previously enrolled to each arm overall, or within a centre or some other prognostic factor, is less than some value *m*, but switching to a biased coin design when the imbalance is *m* or larger.

Response-adaptive allocation is another DA method (Zelen, 1969; Zhou *et al*, 2008). Patients are allocated to treatment as in a biased coin design, that is, allocated to one treatment arm with probability *P*, however, the value of *P* is determined based on the outcomes of patients previously enrolled in the study. If a treatment effect is observed, the value of *P* changes to allow patients a better opportunity of receiving the treatment with the best results. Over the course of the trial, this allocation method aims to optimise patient outcomes and more patients will receive the superior treatment.

## Considerations when selecting an allocation method

A number of points should be considered when selecting an allocation method for use in a particular trial. For example, trials implementing DA methods must have sufficient statistical and programming support available to prevent avoidable algorithm and programming errors. An accessible and reliable centralised database is required for investigators to register patients and to perform the treatment allocation. Depending on the trial, this database may need to be coordinated with centre pharmacies or companies shipping treatment to the study centre. While this support is likely available within most large, cooperative groups, it may be less accessible in smaller centres or companies doing only a limited number of clinical trials. The cost and time required to develop these systems may not be feasible or viable given the small savings in sample size afforded by a balanced trial (Senn, 2004). Alternatively, for very expensive novel therapies, a small savings in sample size could be financially advantageous, particularly if many of the systems are already in place. One might additionally consider potential imbalances in costs between individual study centres if discrepancies were to occur in the number of patients receiving each treatment at different sites. This might occur when there are differences in supportive care costs or in the number of follow-up visits – especially if a costly imaging procedure is included at each follow-up – between treatment arms.

Scientifically, the number and importance of known prognostic factors should factor in the decision of which allocation method to use. If the number and effect on the outcome of prognostic factors is large relative to the total trial sample size, preventing an imbalance might be of greater concern than a trial with few prognostic factors, which has only a modest effect on the outcome and a large sample size. The selection of method to use might be affected if some prognostic factors have a greater effect on outcome than others. Alternatively, one might be concerned with and choose an allocation algorithm based on the issue of selection bias, which might arise in an open-label trial or when the comparison treatments are extremely dissimilar (e.g., surgery *vs* non-surgical therapies). Selection bias arises when investigators can guess with improved probability the treatment future patients will be assigned to receive. Although concerning for deterministic algorithms when physicians know the characteristics of previous patients enrolled, the ability to guess assignment for future patients becomes negligible when centre is not used in the algorithm scheme or a random probability is included in the algorithm (Brown *et al*, 2005).

Finally, one must think about the ultimate analysis and conclusions that might be inferred from a particular trial. Is it likely a sceptic will discount a result if an imbalance is present? Do investigators have sufficient statistical support to address any potential inferential concerns if they are raised? If the trial is being conducted in preparation for a regulatory submission, have the authorities provided any guidance? As recently as 2003, the Committee for Proprietary Medicinal Products noted that DA methods remained highly controversial and strongly advised against their use (Committee for Proprietary Medicinal Products, 2003). Their concern is driven by logistical and practical flaws in previous applications using DA methods, and due to theoretical concerns (Day *et al*, 2005). Although one certainly does not want to use DA methods which could prove problematic if there is no perceived benefit (Day *et al*, 2005), the logistical and practical concerns can be addressed with proper algorithmic testing and attention (Buyse and McEntegart, 2004a). Of greater issue is the theoretical concerns which results from the fact that standard statistical analysis techniques are based on random allocation methods and DA methodology is not random. The distribution of possible outcomes depends on the allocation method used. Consequently, *P*-values obtained using tests which assume random allocation will not be correct when a DA algorithm was used. To illustrate the extent of this potential problem, an example is provided in the next section.

## Illustrative example

Assume eight patients are allocated as part of a clinical trial to one of two treatment arms, as illustrated in Table 2. For simplicity, let the rank order of the patient outcomes be listed and one-sided tests were performed to better illustrate the *P*-value calculations. Two-sided *P*-values could be calculated by doubling the one-sided *P*-value.

Initially, assume one has no knowledge of the prognostic factor status for each patient. At the analysis stage, it is then known that patients 1, 4, 7, and 8 were allocated to treatment arm A, and these patients had the first, sixth, second, and fifth best treatment outcomes. When an incorrect statistical test is applied to these results, the reported *P*-value might be greatly affected. For example, if an investigator ignored that the data were rank order data and assumed the underlying distribution of the data was normal, one might consider using the Welch's two-sample *t*-test, which gives a one-sided *P*-value of 0.1399. An underlying assumption here is that patients in this trial are randomly sampled from the population at large. This assumption may not be true for clinical trials (Ranstam, 2009), although it is often overlooked when allocation of patients to treatment arm is a random process. More appropriately, one might use a permutation test which is not based on the assumption of randomisation. This test proceeds as follows: there are 
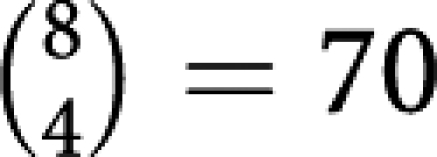
 possible ways of selecting four of the eight patients to receive treatment A. The sum of the ranks of those patients who received treatment A is 14 (1+2+5+6). Of the 70 total permutations, there are 12 for which the sum of ranks is 14 or less; that is, would give as strong, or stronger, evidence in favour of a treatment effect for treatment A. This is the basis for calculating a Wilcoxon's rank-sum test, and gives a one-sided *P*-value of 12/70=0.1714.

Additional information is available given that we know the prognostic factor status of all eight patients. A classical randomisation-based analysis might be to use linear regression, adjusting for the prognostic factor if, similar to the *t*-test, an investigator ignored that the data were rank order data and assumed the underlying distribution of the data was normal. In this case, the one-sided *P*-value is 0.0033. To do a permutation test, one starts with the four prognostic factor-positive patients and calculating the six ways in which these four patients can be allocated such that two patients receive treatment A and two receive treatment B (AABB, ABAB, ABBA, BBAA, BABA, and BAAB). This is similar to using a permuted block allocation method with block size of 4. Of the factor-positive patients, the observed outcome is the most extreme outcome in favour of treatment arm A, since the two patients allocated to arm A (patients 1 and 7) had better outcomes than the two patients allocated to arm B (patients 6 and 3). Therefore, the probability of this (for prognostic factor-positive patients) is 1/6. Similarly, of the factor-negative patients, the two patients allocated to arm A (patients 4 and 8) had better outcomes than the two patients allocated to treatment B (patients 2 and 5). Overall, then, the one-sided *P*-value is 1/6 × 1/6=0.0278.

The *P*-value is different, however, had one used Taves’ deterministic minimisation procedure. Using this algorithm, there are only four allocation possibilities for the factor-positive patients and four possibilities for the factor-negative patients. This is because it is impossible for the first two patients with identical prognostic factor status both to be allocated to the same treatment arm. Patient 1 is allocated to treatment arm A with probability 0.5 which means the next factor-positive patient (patient 3) is deterministically allocated to treatment arm B to minimise the imbalance. The next factor-positive patient (patient 6) is allocated to treatment arm B with probability 0.5, therefore, the next factor-positive patient (patient 7) must receive the opposite treatment (A). Hence, there are only four allocation possibilities: ABAB, ABBA, BABA, and BAAB.

In this situation, patients are paired. Patients 1 and 3 receive opposite treatments, as do patients 6 and 7. Of factor-negative patients, patients 2 and 4 receive opposite treatments, as do patients 5 and 8. In all pairs of patients, the patient who received treatment A did better than the patient who received treatment B. There is no possible way of allocating patients using minimisation, which would create a more extreme outcome favouring treatment A. Then, the one-sided *P*-value is 1/4 × 1/4=0.0625.

Finally, assume one used a biased coin method with *P*=0.8. There remain six possible ways of allocating two of four patients to treatment arm A, within each prognostic factor stratum. However, in this case, the probabilities are different for each possibility, unlike the random permuted blocks method. To calculate the *P*-value, one must calculate the probability of each scenario occurring. The resulting one-sided *P*-value is 0.0434.

In summary, obtaining the correct *P*-value depends on using the correct test for the allocation method which is used (see Table 3). The difference in the *P*-value because of different allocation methods can change the result from a ‘statistically significant at the *α*=0.05 level’ result to a non-statistically significant result.

## Discussion

Despite increasingly frequent implementation of DA methods which aim to balance prognostic factors between treatment arms (Pond *et al*, 2010), their use remains controversial (Senn, 2000). Many of the early criticisms of these methods, that they are too complex, might hinder investigators from performing clinical trials, or that they require a centralised database which might be practically difficult (Peto *et al*, 1976), are less consequential today due to more powerful computers, instant communication, and increasing awareness of the need for comparative clinical trials (Schulz *et al*, 2010). Other criticisms remarking on programming errors can be remedied through vigilance and repeated testing of allocation programs by dedicated statistical and programming teams. Finally, there are concerns that blinding can be compromised when using these methods (Day *et al*, 2005). A number of common strategies can be employed to reduce the possibility of unblinding, such as adding a random component and not using centre as a stratification factor (Brown *et al*, 2005). Importantly, one should not reveal the allocation procedure to investigators involved in enrolling patients and, whenever possible, blind them to the treatment the patients actually receive.

The bigger inferential concern remains, that of using common, but incorrect, statistical analyses which assume random allocation of patients. The use of common statistical tests is in part due to the wide recognition of these tests, but also because it is extremely complex (if not impossible) to perform the correct permutation test when sample sizes, and the number of stratification factors, increase (Knijnenburg *et al*, 2009). One argument for the validity of these tests is that the order of patient accrual can be considered random, although this is not universally accepted (Simon, 1979). Simulations have shown the impact of using random allocation tests instead of permutation tests is small when sample sizes are reasonably large and adjustment for prognostic factors is performed (Birkett, 1985; Kalish and Begg, 1987; Tu *et al*, 2000); thus, the use of standard tests should not be much of a concern (McEntegart, 2003). It is further noted that use of statistical tests which assume random allocation are frequently applied when using permuted block methods, another non-random procedure, and there is little concern of the impact on these results.

While most authors advocate adjusting for covariates used as stratification factors in the final analysis, the argument of increased credibility of DA methods over simple random sampling is related to the unadjusted, univariate model (Buyse and McEntegart, 2004a). As the presented example shows there might be substantial differences between the unadjusted and adjusted analyses, and it is always important to investigate when there are differences.

Numerous options are available to investigators conducting a cancer clinical trial for treatment allocation between comparison arms. Careful consideration should occur in the trial design phase to select the method best suited for a given trial. This decision could impact the inferential ability and credibility of a trial and should not be perceived as trivial. Knowledge of the impact of the allocation method on the trial is essential to proper understanding of the results, regardless of procedure used.

In summary, DA should be considered a valid alternative to randomisation or allocation via the random permuted blocks method, particularly for small to moderate-sized clinical trials with multiple significant prognostic factors having modest to large treatment effects, as is common in oncology. While it is recommended that only a few factors be used when using the random permuted blocks method – as a rule of thumb, the total number of cells should be less than *n*/2 – DA methods can handle many factors without difficulty (Therneau, 1993). Even with DA methods, however, it is advised that only factors with a known, large prognostic effect be included and there should be at least five patients per cell (Rovers *et al*, 2000). Using a balanced coin algorithm and incorporating a random element with probability *P=0.8* is most efficient for reducing the ability to predict future patient allocations while maintaining good balance (Brown *et al*, 2005). While statistical tests based on the assumption of randomisation may give similar results, the *P*-value will only be precisely correct when using the statistical test corresponding to the allocation algorithm used. At a minimum, investigators should perform multivariable analyses which adjust for all factors used in the DA algorithm and perform appropriate sensitivity analyses (Committee for Proprietary Medicinal Products, 2003).

## Figures and Tables

**Table 1:** Summary of baseline prognostic factors for first 19 patients on hypothetical trial

		**Treatment A**	**Treatment B**
Number of patients		10	9
Her2-neu status	*Positive*:*Negative*	5 : 5	6 : 3
Menopausal status	*Pre-/peri–post-*:	4 : 6	5 : 4
Stage	*II:III*	7 : 3	2 : 7

**Table 2:** Hypothetical clinical trial

**Order of patient entry into study**	**Treatment allocation**	**Prognostic factor**	**Outcome order**
1	A	+	1
2	B	−	8
3	B	+	4
4	A	−	6
5	B	−	7
6	B	+	3
7	A	+	2
8	A	−	5

**Table 3:** *P*-values based on allocation method

**No prognostic factor information**		**Incorporating prognostic factor information**	
**Allocation method**	**One-sided *P*-value**	**Allocation method**	**One-sided *P*-value**
Random sampling	0.1399	Random sampling	0.0033
Permutation test	0.1714	Random permuted blocks	0.0278
		Deterministic minimisation	0.0625
		Biased coin	0.0434
